# Supplementary material for: Smooth Interpolating Curves with Local Control and Monotone Alternating Curvature
Source: Comput Graph Forum. 2022 Oct 6;41(5):25–38. doi: 10.1111/cgf.14600 (PMC9827861; doi:10.1111/cgf.14600)
Supplement: Supplementary file 1 — Supplement Material [file CGF-41-25-s001.zip › Local-Smooth-Interpolating-MonoCurvature/extern/clothoids/docs/api-cpp/program_listing_file_PolyLine.cc.html]

Program Listing for File PolyLine.cc — Clothoids v2.0.9

### Navigation

- index
- toc
- Clothoids »
- Program Listing for File PolyLine.cc

# Program Listing for File PolyLine.cc¶

↰ Return to documentation for file (`PolyLine.cc`)

```
/*--------------------------------------------------------------------------*\
 |                                                                          |
 |  Copyright (C) 2017                                                      |
 |                                                                          |
 |         , __                 , __                                        |
 |        /|/  \               /|/  \                                       |
 |         | __/ _   ,_         | __/ _   ,_                                |
 |         |   \|/  /  |  |   | |   \|/  /  |  |   |                        |
 |         |(__/|__/   |_/ \_/|/|(__/|__/   |_/ \_/|/                       |
 |                           /|                   /|                        |
 |                           \|                   \|                        |
 |                                                                          |
 |      Enrico Bertolazzi                                                   |
 |      Dipartimento di Ingegneria Industriale                              |
 |      Universita` degli Studi di Trento                                   |
 |      email: enrico.bertolazzi@unitn.it                                   |
 |                                                                          |
\*--------------------------------------------------------------------------*/

#include "Clothoids.hh"

// Workaround for Visual Studio
#ifdef min
  #undef min
#endif

#ifdef max
  #undef max
#endif

#include <algorithm>

namespace G2lib {

  using std::min;
  using std::max;
  using std::abs;
  using std::cout;
  using std::vector;
  using std::ceil;

  #ifndef DOXYGEN_SHOULD_SKIP_THIS
  typedef vector<LineSegment>::difference_type LS_dist_type;
  #endif

  /*\
   |  ____       _       _     _
   | |  _ \ ___ | |_   _| |   (_)_ __   ___
   | | |_) / _ \| | | | | |   | | '_ \ / _ \
   | |  __/ (_) | | |_| | |___| | | | |  __/
   | |_|   \___/|_|\__, |_____|_|_| |_|\___|
   |               |___/
  \*/

  // . . . . . . . . . . . . . . . . . . . . . . . . . . . . . . . . . . . . .

  PolyLine::PolyLine( BaseCurve const & C )
  : BaseCurve(G2LIB_POLYLINE)
  , m_aabb_done(false)
  {
    this->resetLastInterval();
    switch ( C.type() ) {
    case G2LIB_LINE:
      build( *static_cast<LineSegment const *>(&C) );
      break;
    case G2LIB_POLYLINE:
      copy( *static_cast<PolyLine const *>(&C) );
      break;
    case G2LIB_CIRCLE:
    case G2LIB_CLOTHOID:
    case G2LIB_BIARC:
    case G2LIB_BIARC_LIST:
    case G2LIB_CLOTHOID_LIST:
      UTILS_ERROR(
        "PolyLine constructor cannot convert from: {}\n",
        CurveType_name[C.type()]
      );
    }
  }

  // - - - - - - - - - - - - - - - - - - - - - - - - - - - - - - - - - - - - - -

  PolyLine::PolyLine( LineSegment const & LS )
  : BaseCurve(G2LIB_POLYLINE)
  , m_aabb_done(false)
  {
    this->resetLastInterval();
    this->init( LS.xBegin(), LS.xBegin() );
    this->push_back( LS );
  }

  // - - - - - - - - - - - - - - - - - - - - - - - - - - - - - - - - - - - - - -

  PolyLine::PolyLine( CircleArc const & C, real_type tol )
  : BaseCurve(G2LIB_POLYLINE)
  , m_aabb_done(false)
  {
    this->resetLastInterval();
    this->init( C.xBegin(), C.xBegin() );
    this->push_back( C, tol );
  }

  // - - - - - - - - - - - - - - - - - - - - - - - - - - - - - - - - - - - - - -

  PolyLine::PolyLine( Biarc const & B, real_type tol )
  : BaseCurve(G2LIB_POLYLINE)
  , m_aabb_done(false)
  {
    this->resetLastInterval();
    this->init( B.xBegin(), B.xBegin() );
    this->push_back( B, tol );
  }

  // - - - - - - - - - - - - - - - - - - - - - - - - - - - - - - - - - - - - - -

  PolyLine::PolyLine( ClothoidCurve const & C, real_type tol )
  : BaseCurve(G2LIB_POLYLINE)
  , m_aabb_done(false)
  {
    this->resetLastInterval();
    this->init( C.xBegin(), C.xBegin() );
    this->push_back( C, tol );
  }

  // - - - - - - - - - - - - - - - - - - - - - - - - - - - - - - - - - - - - - -

  PolyLine::PolyLine( ClothoidList const & PL, real_type tol )
  : BaseCurve(G2LIB_POLYLINE)
  , m_aabb_done(false)
  {
    this->resetLastInterval();
    this->init( PL.xBegin(), PL.xBegin() );
    this->push_back( PL, tol );
  }

  // - - - - - - - - - - - - - - - - - - - - - - - - - - - - - - - - - - - - - -

  int_type
  PolyLine::findAtS( real_type & s ) const {
    bool ok;
    int_type & lastInterval = *m_lastInterval.search( std::this_thread::get_id(), ok );
    Utils::searchInterval<int_type,real_type>(
      static_cast<int_type>(m_s0.size()),
      &m_s0.front(), s, lastInterval, false, true
    );
    return lastInterval;
  }

  // - - - - - - - - - - - - - - - - - - - - - - - - - - - - - - - - - - - - - -

  void
  PolyLine::init() {
    m_s0.clear();
    m_polylineList.clear();
    this->resetLastInterval();
    m_aabb_done = false;
    this->resetLastInterval();
  }

  // - - - - - - - - - - - - - - - - - - - - - - - - - - - - - - - - - - - - - -

  void
  PolyLine::copy( PolyLine const & PL ) {
    this->init();
    m_polylineList.reserve( PL.m_polylineList.size() );
    std::copy(
      PL.m_polylineList.begin(),
      PL.m_polylineList.end(),
      back_inserter(m_polylineList)
    );
    m_s0.reserve( PL.m_s0.size() );
    std::copy( PL.m_s0.begin(), PL.m_s0.end(), back_inserter(m_s0) );
  }

  // - - - - - - - - - - - - - - - - - - - - - - - - - - - - - - - - - - - - - -

  LineSegment const &
  PolyLine::getSegment( int_type n ) const {
    UTILS_ASSERT0(
      !m_polylineList.empty(),
      "PolyLine::getSegment(...) empty PolyLine\n"
    );
    UTILS_ASSERT(
      n >= 0 && n < int_type(m_polylineList.size()),
      "PolyLine::getSegment( {} ) out of range [0,{}]\n",
      n, m_polylineList.size()-1
    );
    return m_polylineList[size_t(n)];
  }

  // - - - - - - - - - - - - - - - - - - - - - - - - - - - - - - - - - - - - - -

  void
  PolyLine::polygon( real_type * x, real_type * y) const {
    int_type n = int_type(m_polylineList.size());
    for ( size_t k = 0; k < size_t(n); ++k ) {
      x[k] = m_polylineList[k].xBegin();
      y[k] = m_polylineList[k].yBegin();
    }
    x[size_t(n)] = m_polylineList[size_t(n-1)].xEnd();
    y[size_t(n)] = m_polylineList[size_t(n-1)].yEnd();
  }

  /*\
   |   _     _
   |  | |__ | |__   _____  __
   |  | '_ \| '_ \ / _ \ \/ /
   |  | |_) | |_) | (_) >  <
   |  |_.__/|_.__/ \___/_/\_\
  \*/

  // - - - - - - - - - - - - - - - - - - - - - - - - - - - - - - - - - - - - - -

  void
  PolyLine::bbox(
    real_type & xmin,
    real_type & ymin,
    real_type & xmax,
    real_type & ymax
  ) const {

    UTILS_ASSERT0( !m_polylineList.empty(), "PolyLine::bbox, empty list\n" );

    if ( m_aabb_done ) {
      m_aabb_tree.bbox( xmin, ymin, xmax, ymax );
    } else {
      vector<LineSegment>::const_iterator ic = m_polylineList.begin();
      xmin = xmax = ic->xBegin();
      ymin = ymax = ic->yBegin();
      for ( ++ic; ic != m_polylineList.end(); ++ic ) {
        real_type x = ic->xBegin();
        real_type y = ic->yBegin();
        if      ( x < xmin ) xmin = x;
        else if ( x > xmax ) xmax = x;
        if      ( y < ymin ) ymin = y;
        else if ( y > ymax ) ymax = y;
      }
      --ic;
      real_type x = ic->xEnd();
      real_type y = ic->yEnd();
      if      ( x < xmin ) xmin = x;
      else if ( x > xmax ) xmax = x;
      if      ( y < ymin ) ymin = y;
      else if ( y > ymax ) ymax = y;
    }
  }

  /*\
   |  _    _   _____    _                _
   | | |__| |_|_   _| _(_)__ _ _ _  __ _| |___
   | | '_ \ '_ \| || '_| / _` | ' \/ _` | / -_)
   | |_.__/_.__/|_||_| |_\__,_|_||_\__, |_\___|
   |                               |___/
  \*/

  void
  PolyLine::bbTriangles(
    vector<Triangle2D> & tvec,
    real_type            max_angle,
    real_type            max_size,
    int_type             icurve
  ) const {
    vector<LineSegment>::const_iterator ic = m_polylineList.begin();
    for ( int_type ipos = icurve; ic != m_polylineList.end(); ++ic, ++ipos )
      ic->bbTriangles( tvec, max_angle, max_size, ipos );
  }

  // . . . . . . . . . . . . . . . . . . . . . . . . . . . . . . . . . . . . .

  void
  PolyLine::bbTriangles_ISO(
    real_type            offs,
    vector<Triangle2D> & tvec,
    real_type            max_angle,
    real_type            max_size,
    int_type             icurve
  ) const {
    vector<LineSegment>::const_iterator ic = m_polylineList.begin();
    for ( int_type ipos = icurve; ic != m_polylineList.end(); ++ic, ++ipos )
      ic->bbTriangles_ISO( offs, tvec, max_angle, max_size, ipos );
  }

  // - - - - - - - - - - - - - - - - - - - - - - - - - - - - - - - - - - - - - -

  real_type
  PolyLine::theta( real_type s ) const {
    int_type idx = this->findAtS( s );
    return m_polylineList[idx].m_theta0;
  }

  // - - - - - - - - - - - - - - - - - - - - - - - - - - - - - - - - - - - - - -

  real_type
  PolyLine::theta_D( real_type ) const
  { return 0; }

  // - - - - - - - - - - - - - - - - - - - - - - - - - - - - - - - - - - - - - -

  real_type
  PolyLine::theta_DD( real_type ) const
  { return 0; }

  // - - - - - - - - - - - - - - - - - - - - - - - - - - - - - - - - - - - - - -

  real_type
  PolyLine::theta_DDD( real_type ) const
  { return 0; }

  /*\
   |  _                        __
   | | |_ _ __ __ _ _ __  ___ / _| ___  _ __ _ __ ___
   | | __| '__/ _` | '_ \/ __| |_ / _ \| '__| '_ ` _ \
   | | |_| | | (_| | | | \__ \  _| (_) | |  | | | | | |
   |  \__|_|  \__,_|_| |_|___/_|  \___/|_|  |_| |_| |_|
  \*/

  // - - - - - - - - - - - - - - - - - - - - - - - - - - - - - - - - - - - - - -

  void
  PolyLine::scale( real_type sfactor ) {
    vector<LineSegment>::iterator ic = m_polylineList.begin();
    real_type newx0 = ic->xBegin();
    real_type newy0 = ic->yBegin();
    m_s0[0] = 0;
    for ( size_t k=0; ic != m_polylineList.end(); ++ic, ++k ) {
      ic->scale( sfactor );
      ic->changeOrigin( newx0, newy0 );
      newx0     = ic->xEnd();
      newy0     = ic->yEnd();
      m_s0[k+1] = m_s0[k] + ic->length();
    }
  }

  // - - - - - - - - - - - - - - - - - - - - - - - - - - - - - - - - - - - - - -

  void
  PolyLine::reverse() {
    std::reverse( m_polylineList.begin(), m_polylineList.end() );
    vector<LineSegment>::iterator ic = m_polylineList.begin();
    ic->reverse();
    real_type newx0 = ic->xEnd();
    real_type newy0 = ic->yEnd();
    m_s0[0] = 0;
    m_s0[1] = ic->length();
    size_t k = 1;
    for ( ++ic; ic != m_polylineList.end(); ++ic, ++k ) {
      ic->reverse();
      ic->changeOrigin( newx0, newy0 );
      newx0     = ic->xEnd();
      newy0     = ic->yEnd();
      m_s0[k+1] = m_s0[k] + ic->length();
    }
  }

  // - - - - - - - - - - - - - - - - - - - - - - - - - - - - - - - - - - - - - -

  void
  PolyLine::changeOrigin( real_type newx0, real_type newy0 ) {
    vector<LineSegment>::iterator ic = m_polylineList.begin();
    for (; ic != m_polylineList.end(); ++ic ) {
      ic->changeOrigin( newx0, newy0 );
      newx0 = ic->xEnd();
      newy0 = ic->yEnd();
    }
  }

  // - - - - - - - - - - - - - - - - - - - - - - - - - - - - - - - - - - - - - -

  void
  PolyLine::trim( real_type s_begin, real_type s_end ) {
    UTILS_ASSERT(
      s_begin >= m_s0.front() && s_end <= m_s0.back() && s_end > s_begin,
      "void::trim( s_begin={}, s_end={} ) bad range, must be in [{},{}]\n",
      s_begin, s_end, m_s0.front(), m_s0.back()
    );

    size_t i_begin = size_t(findAtS(s_begin));
    size_t i_end   = size_t(findAtS(s_end));
    m_polylineList[i_begin].trim( s_begin-m_s0[i_begin], m_s0[i_begin+1] );
    m_polylineList[i_end].trim( m_s0[i_end], s_end-m_s0[i_end] );
    m_polylineList.erase( m_polylineList.begin()+LS_dist_type(i_end+1), m_polylineList.end() );
    m_polylineList.erase( m_polylineList.begin(), m_polylineList.begin()+LS_dist_type(i_begin) );
    vector<LineSegment>::iterator ic = m_polylineList.begin();
    m_s0[0] = 0;
    size_t k = 0;
    for (; ic != m_polylineList.end(); ++ic, ++k )
      m_s0[k+1] = m_s0[k] + ic->length();
    this->resetLastInterval();
  }

  // - - - - - - - - - - - - - - - - - - - - - - - - - - - - - - - - - - - - - -

  void
  PolyLine::trim( real_type s_begin, real_type s_end, PolyLine & newPL ) const {

    newPL.init();

    if ( m_polylineList.empty() ) return;

    // put in range
    real_type L = this->length();
    while ( s_begin > L ) s_begin -= L;
    while ( s_begin < 0 ) s_begin += L;
    while ( s_end   > L ) s_end   -= L;
    while ( s_end   < 0 ) s_end   += L;

    int_type n_seg   = int_type( m_polylineList.size() );
    int_type i_begin = findAtS( s_begin );
    int_type i_end   = findAtS( s_end );

    if ( s_begin < s_end ) {
      // get initial and final segment
      if ( i_begin == i_end ) { // stesso segmento
        real_type   ss0 = m_s0[i_begin];
        LineSegment LL  = m_polylineList[i_begin];
        LL.trim( s_begin-ss0, s_end-ss0 );
        newPL.push_back( LL );
      } else {
        LineSegment L0 = m_polylineList[i_begin];
        L0.trim( s_begin - m_s0[i_begin], L0.length() );
        newPL.push_back( L0 );

        for ( ++i_begin; i_begin < i_end; ++i_begin )
          newPL.push_back( m_polylineList[i_begin] );

        LineSegment L1 = m_polylineList[i_end];
        L1.trim( 0, s_end - m_s0[i_end] );
        newPL.push_back( L1 );
      }
    } else {
      LineSegment L0 = m_polylineList[i_begin];
      L0.trim( s_begin - m_s0[i_begin], L0.length() );
      newPL.push_back( L0 );

      for ( ++i_begin; i_begin < n_seg; ++i_begin )
        newPL.push_back( m_polylineList[i_begin] );

      for ( i_begin = 0; i_begin < i_end; ++i_begin )
        newPL.push_back( m_polylineList[i_begin] );

      LineSegment L1 = m_polylineList[i_end];
      L1.trim( 0, s_end - m_s0[i_end] );
      newPL.push_back( L1 );
    }
  }

  // - - - - - - - - - - - - - - - - - - - - - - - - - - - - - - - - - - - - - -

  void
  PolyLine::build_AABBtree( AABBtree & aabbtree ) const {
    #ifdef G2LIB_USE_CXX11
    vector<shared_ptr<BBox const> > bboxes;
    #else
    vector<BBox const *> bboxes;
    #endif
    bboxes.reserve(m_polylineList.size());
    vector<LineSegment>::const_iterator it;
    int_type ipos = 0;
    for ( it = m_polylineList.begin(); it != m_polylineList.end(); ++it, ++ipos ) {
      real_type xmin, ymin, xmax, ymax;
      it->bbox( xmin, ymin, xmax, ymax );
      #ifdef G2LIB_USE_CXX11
      bboxes.push_back( make_shared<BBox const>(
        xmin, ymin, xmax, ymax, G2LIB_LINE, ipos
      ) );
      #else
      bboxes.push_back( new BBox( xmin, ymin, xmax, ymax, G2LIB_LINE, ipos ) );
      #endif
    }
    aabbtree.build(bboxes);
  }

  // - - - - - - - - - - - - - - - - - - - - - - - - - - - - - - - - - - - - - -

  void
  PolyLine::init( real_type x0, real_type y0 ) {
    m_xe = x0;
    m_ye = y0;
    m_polylineList.clear();
    m_s0.clear();
    m_s0.push_back(0);
    m_aabb_done = false;
  }

  // - - - - - - - - - - - - - - - - - - - - - - - - - - - - - - - - - - - - - -

  void
  PolyLine::push_back( real_type x, real_type y ) {
    LineSegment s;
    s.build_2P( m_xe, m_ye, x, y );
    m_polylineList.push_back( s );
    real_type slast = m_s0.back() + s.length();
    m_s0.push_back( slast );
    m_xe = x;
    m_ye = y;
    m_aabb_done = false;
  }

  // - - - - - - - - - - - - - - - - - - - - - - - - - - - - - - - - - - - - - -

  void
  PolyLine::push_back( LineSegment const & C ) {
    m_polylineList.push_back( C );
    LineSegment & S = m_polylineList.back();
    S.changeOrigin( m_xe, m_ye );
    real_type slast = m_s0.back() + S.length();
    m_s0.push_back( slast );
    m_xe = S.xEnd();
    m_ye = S.yEnd();
    m_aabb_done = false;
  }

  // - - - - - - - - - - - - - - - - - - - - - - - - - - - - - - - - - - - - - -

  void
  PolyLine::push_back( CircleArc const & C, real_type tol ) {
    real_type L  = C.length();
    int_type  ns = int_type(ceil( L / C.lenTolerance( tol ) ));
    real_type tx = m_xe - C.xBegin();
    real_type ty = m_ye - C.yBegin();
    for ( int_type i = 1; i < ns; ++i ) {
      real_type s = (i*L)/ns;
      push_back( tx + C.X(s), ty + C.Y(s) );
    }
    push_back( tx + C.xEnd(), ty + C.yEnd() );
    m_xe = tx + C.xEnd();
    m_ye = ty + C.yEnd();
    m_aabb_done = false;
  }

  // - - - - - - - - - - - - - - - - - - - - - - - - - - - - - - - - - - - - - -

  void
  PolyLine::push_back( Biarc const & B, real_type tol ) {
    CircleArc const & C0 = B.C0();
    CircleArc const & C1 = B.C1();
    real_type L0  = C0.length();
    real_type L1  = C1.length();
    int_type  ns0 = int_type(ceil( L0 / C0.lenTolerance( tol ) ));
    int_type  ns1 = int_type(ceil( L1 / C1.lenTolerance( tol ) ));

    real_type tx = m_xe - C0.xBegin();
    real_type ty = m_ye - C0.yBegin();

    for ( int_type i = 1; i < ns0; ++i ) {
      real_type s = (i*L0)/ns0;
      push_back( tx + C0.X(s), ty + C0.Y(s) );
    }
    push_back( tx + C1.xBegin(), ty + C1.yBegin() );
    for ( int_type i = 1; i < ns1; ++i ) {
      real_type s = (i*L1)/ns1;
      push_back( tx + C1.X(s), ty + C1.Y(s) );
    }
    push_back( tx + C1.xEnd(), ty + C1.yEnd() );
    m_xe = tx + C1.xEnd();
    m_ye = ty + C1.yEnd();
    m_aabb_done = false;
  }

  // - - - - - - - - - - - - - - - - - - - - - - - - - - - - - - - - - - - - - -

  void
  PolyLine::push_back( ClothoidCurve const & C, real_type tol ) {

    real_type L    = C.length();
    real_type absk = max(abs(C.kappaBegin()), abs(C.kappaEnd()));
    real_type tmp  = absk*tol - 1;
    int_type ns = 1;
    if ( tmp > -1 ) ns = int_type( ceil( L*absk/(2*(Utils::m_pi-acos(tmp))) ) );

    real_type tx = m_xe - C.xBegin();
    real_type ty = m_ye - C.yBegin();
    for ( int_type i = 1; i < ns; ++i ) {
      real_type s = (i*L)/ns;
      push_back( tx + C.X(s), ty + C.Y(s) );
    }

    push_back( tx + C.xEnd(), ty + C.yEnd() );
    m_xe = tx + C.xEnd();
    m_ye = ty + C.yEnd();
    m_aabb_done = false;
  }

  // - - - - - - - - - - - - - - - - - - - - - - - - - - - - - - - - - - - - - -

  void
  PolyLine::push_back( ClothoidList const & L, real_type tol ) {
    int_type ns = L.numSegments();
    for ( int_type idx = 0; idx < ns; ++idx ) {
      ClothoidCurve const & C = L.get( idx );
      push_back( C, tol );
    }
    // aabb_done = false;
  }

  // - - - - - - - - - - - - - - - - - - - - - - - - - - - - - - - - - - - - - -

  void
  PolyLine::build(
    real_type const * x,
    real_type const * y,
    int_type          npts
  ) {
    init( x[0], y[0] );
    for ( int_type k = 1; k < npts; ++k )
      push_back( x[k], y[k] );
  }

  // - - - - - - - - - - - - - - - - - - - - - - - - - - - - - - - - - - - - - -

  void
  PolyLine::build( LineSegment const & C ) {
    init( C.xBegin(), C.yBegin() );
    push_back( C.xEnd(), C.yEnd() );
  }

  // - - - - - - - - - - - - - - - - - - - - - - - - - - - - - - - - - - - - - -

  void
  PolyLine::build( CircleArc const & C, real_type tol ) {
    init( C.xBegin(), C.yBegin() );
    push_back( C, tol );
  }

  // - - - - - - - - - - - - - - - - - - - - - - - - - - - - - - - - - - - - - -

  void
  PolyLine::build( Biarc const & C, real_type tol ) {
    init( C.xBegin(), C.yBegin() );
    push_back( C, tol );
  }

  // - - - - - - - - - - - - - - - - - - - - - - - - - - - - - - - - - - - - - -

  void
  PolyLine::build( ClothoidCurve const & C, real_type tol ) {
    init( C.xBegin(), C.yBegin() );
    push_back( C, tol );
  }

  // - - - - - - - - - - - - - - - - - - - - - - - - - - - - - - - - - - - - - -

  void
  PolyLine::build( ClothoidList const & L, real_type tol ) {
    init( L.xBegin(), L.yBegin() );
    push_back( L, tol );
  }

  // - - - - - - - - - - - - - - - - - - - - - - - - - - - - - - - - - - - - - -

  int_type
  PolyLine::closestPoint_ISO(
    real_type   x,
    real_type   y,
    real_type & X,
    real_type & Y,
    real_type & S,
    real_type & T,
    real_type & DST
  ) const{
    UTILS_ASSERT0(
      !m_polylineList.empty(),
      "PolyLine::closestPoint, empty list\n"
    );
    vector<LineSegment>::const_iterator ic = m_polylineList.begin();
    vector<real_type>::const_iterator   is = m_s0.begin();
    ic->closestPoint_ISO( x, y, X, Y, S, T, DST );
    int_type ipos = 0;
    for ( ++ic, ++is; ic != m_polylineList.end(); ++ic, ++is ) {
      real_type X1, Y1, S1, T1, DST1;
      ic->closestPoint_ISO( x, y, X1, Y1, S1, T1, DST1 );
      if ( DST1 < DST ) {
        DST  = DST1;
        X    = X1;
        Y    = Y1;
        S    = *is + S1;
        T    = T1;
        ipos = int_type(ic-m_polylineList.begin());
      }
    }

    real_type xx, yy;
    m_polylineList[size_t(ipos)].eval_ISO( S - m_s0[size_t(ipos)], T, xx, yy );
    real_type err = hypot( x - xx, y - yy );
    real_type tol = (DST > 1 ? DST*machepsi1000 : machepsi1000);
    if ( err > tol ) return -(ipos+1);
    return ipos;
  }

  /*\
   |             _ _ _     _
   |    ___ ___ | | (_)___(_) ___  _ __
   |   / __/ _ \| | | / __| |/ _ \| '_ \
   |  | (_| (_) | | | \__ \ | (_) | | | |
   |   \___\___/|_|_|_|___/_|\___/|_| |_|
  \*/

  bool
  PolyLine::collision( PolyLine const & C ) const {
    this->build_AABBtree();
    C.build_AABBtree();
    Collision_list fun( this, &C );
    return m_aabb_tree.collision( C.m_aabb_tree, fun, false );
  }

  /*\
   |   _       _                          _
   |  (_)_ __ | |_ ___ _ __ ___  ___  ___| |_
   |  | | '_ \| __/ _ \ '__/ __|/ _ \/ __| __|
   |  | | | | | ||  __/ |  \__ \  __/ (__| |_
   |  |_|_| |_|\__\___|_|  |___/\___|\___|\__|
  \*/

  void
  PolyLine::intersect(
    PolyLine const    & pl,
    vector<real_type> & ss0,
    vector<real_type> & ss1
  ) const {
    UTILS_ASSERT0(
      !m_polylineList.empty(),
      "PolyLine::intersect, empty list\n"
    );
    UTILS_ASSERT(
      !pl.m_polylineList.empty(),
      "PolyLine::intersect, empty secondary list\n"
    );

#if 1
    build_AABBtree();
    pl.build_AABBtree();
    AABBtree::VecPairPtrBBox intersectionList;
    m_aabb_tree.intersect( pl.m_aabb_tree, intersectionList );
    AABBtree::VecPairPtrBBox::const_iterator ip;
    for ( ip = intersectionList.begin(); ip != intersectionList.end(); ++ip ) {
      size_t ipos0 = size_t(ip->first->Ipos());
      size_t ipos1 = size_t(ip->second->Ipos());
      UTILS_ASSERT(
        ipos0 < m_polylineList.size(),
        "Bad ipos0 = {}\n", ipos0
      );
      UTILS_ASSERT(
        ipos1 < pl.m_polylineList.size(),
        "Bad ipos1 = {}\n", ipos1
      );
      real_type sss0, sss1;
      bool ok = m_polylineList[ipos0].intersect(pl.m_polylineList[ipos1],sss0,sss1);
      if ( ok ) {
        ss0.push_back(sss0+m_s0[ipos0]);
        ss1.push_back(sss1+pl.m_s0[ipos1]);
      }
    }

#else
    ss0.clear();
    ss1.clear();
    vector<LineSegment>::const_iterator ic0 = lvec.begin();
    vector<real_type>::const_iterator   is0 = s0.begin();
    while ( ic0 != lvec.end() ) {
      vector<LineSegment>::const_iterator ic1 = pl.lvec.begin();
      vector<real_type>::const_iterator   is1 = pl.s0.begin();
      while ( ic1 != pl.lvec.end() ) {
        real_type a0, a1;
        bool ok = ic0->intersect( *ic1, a0, a1 );
        if ( ok ) {
          ss0.push_back( (*is0) + a0 );
          ss1.push_back( (*is1) + a1 );
        }
        ++ic1;
        ++is1;
      }
      ++ic0;
      ++is0;
    }
#endif

  }

  void
  PolyLine::intersect(
    PolyLine const & pl,
    IntersectList  & ilist,
    bool             swap_s_vals
  ) const {
    std::vector<real_type> s1, s2;
    this->intersect( pl, s1, s2 );
    ilist.reserve( ilist.size() + s1.size() );
    for ( size_t i=0; i < s1.size(); ++i ) {
      real_type ss1 = s1[i];
      real_type ss2 = s2[i];
      if ( swap_s_vals ) std::swap( ss1, ss2 );
      ilist.push_back( Ipair(ss1,ss2) );
    }
  }

  // - - - - - - - - - - - - - - - - - - - - - - - - - - - - - - - - - - - - - -

  ostream_type &
  operator << ( ostream_type & stream, PolyLine const & P ) {
    fmt::print( stream,
      "nseg   = {}\n"
      "xBegin = {}\n"
      "ybegin = {}\n"
      "xEnd   = {}\n"
      "yEnd   = {}\n"
      "length = {}\n",
      P.numSegments(),
      P.xBegin(),
      P.yBegin(),
      P.xEnd(),
      P.yEnd(),
      P.length()
    );
    return stream;
  }

  // - - - - - - - - - - - - - - - - - - - - - - - - - - - - - - - - - - - - - -

}

// EOF: PolyLine.cc
```

### Quick search

### Table of Contents

- Matlab Interface Manual
- C++ API
- MATLAB API

«
hide menu

menu
sidebar
»

### Navigation

- index
- toc
- Clothoids »
- Program Listing for File PolyLine.cc

© Copyright 2021, Enrico Bertolazzi and Marco Frego.
Created using Sphinx 4.2.0.
